# The Investment Development Path theory: Evidence from developing countries’ agricultural sector

**DOI:** 10.12688/f1000research.139491.1

**Published:** 2023-09-28

**Authors:** Justice Gameli Djokoto, Kofi Aaron A-O Agyei-Henaku, Charlotte Badu-Prah

**Affiliations:** 1 Agribusiness Management, Central University, Accra, Greater Accra Region, Ghana

**Keywords:** Agriculture, developing countries, IDP, internalisation advantage, inward foreign direct investment, investment development path, location advantage, ownership advantage, outward foreign direct investment

## Abstract

**Background:** We examined the investment development path (IDP) through the perspective of developing countries’ agricultural sector. Our analytical approach indirectly accounts for interactions among countries regarding cross-border resource transfers. Aside from providing knowledge on testing the IDP by inferential statistics, the information would be relevant for policymaking. Identifying the stage(s) in the IDP not only highlights the global appeal of agriculture but also guides firms seeking to expand beyond borders. This information is essential for developing an effective economic strategy.

**Methods:** We employed data from 1991 to 2021 for 55 countries from the Food and Agriculture Organization Corporate Statistical Database (FAOSTAT) and applied a fixed effects estimator corrected for serial correlation and non-constant variances.

**Results and conclusions:** We found that agriculture in developing countries is currently in stages I and II of the IDP. Broadly, agricultural production requires policies that would increase outward foreign direct investment and inward foreign direct investment. Domestic agricultural businesses in developing countries must develop capacity by learning from foreign multinationals. This would enable agricultural businesses to invest abroad. Such a move would lead to an increase in outward FDI. As this would have resulted from increased GDP per capita, it will lead to movement from the existing stage to higher ones.

## Introduction

The net outward and direct investment situation of a country methodically correlates with its economic progress ( Dunning, 1981a). Stages I, II, III, IV and V have been identified. These reflect the international attractiveness of the economy and firms’ cross-border expansion and give guiding steps for an appropriate economic strategy ( Buckley and Castro, 1998; Djokoto and Pomeyie, 2021; Dunning, 1981a, b, 1986; Dunning and Narula, 1996; Gorynia *et al.*, 2019).

As in the case of the total economy, the IDP could apply to the agriculture sector which continues to be important for many developing countries. The World Bank notes that developing agriculture is an extremely effective instrument to stop severe lack, promote collective wealth and feed an anticipated 9.7 billion persons by the year 2050. Compared to other sectors, the growth of the agricultural sector is two and four times more operative in boosting incomes amongst the most deprived ( World Bank, 2022). Thus, developing countries have sought agricultural development through policies to entice foreign direct investment (FDI) into the agricultural sector ( Djokoto, 2022; Ju *et al.*, 2022; Tian, 2023; Wardhani and Haryanto, 2020). As a result of knowledge gained from foreign firms, agricultural multinationals (AM) have emerged in some developing countries ( Chen and Guo, 2017). The outcome of the FDI policies and the progress of the AMs from developing countries have resulted in both inward and outward FDI into and out of developing countries ( Chen and Guo, 2017; Ju *et al.*, 2022; Tian, 2023; Wardhani and Haryanto, 2020). Indeed, from 2008 to 2019, $68.7 billion and $63.75 billion respectively were recorded as inward and outward FDI into agriculture. This is within the global investment need of $5 to $7 trillion per year ( United Nations, 2014). How does the interaction between the inward and the outward FDI on one hand and agricultural development on the other explain the level of development in developing countries’ agriculture?

**Figure 1.**
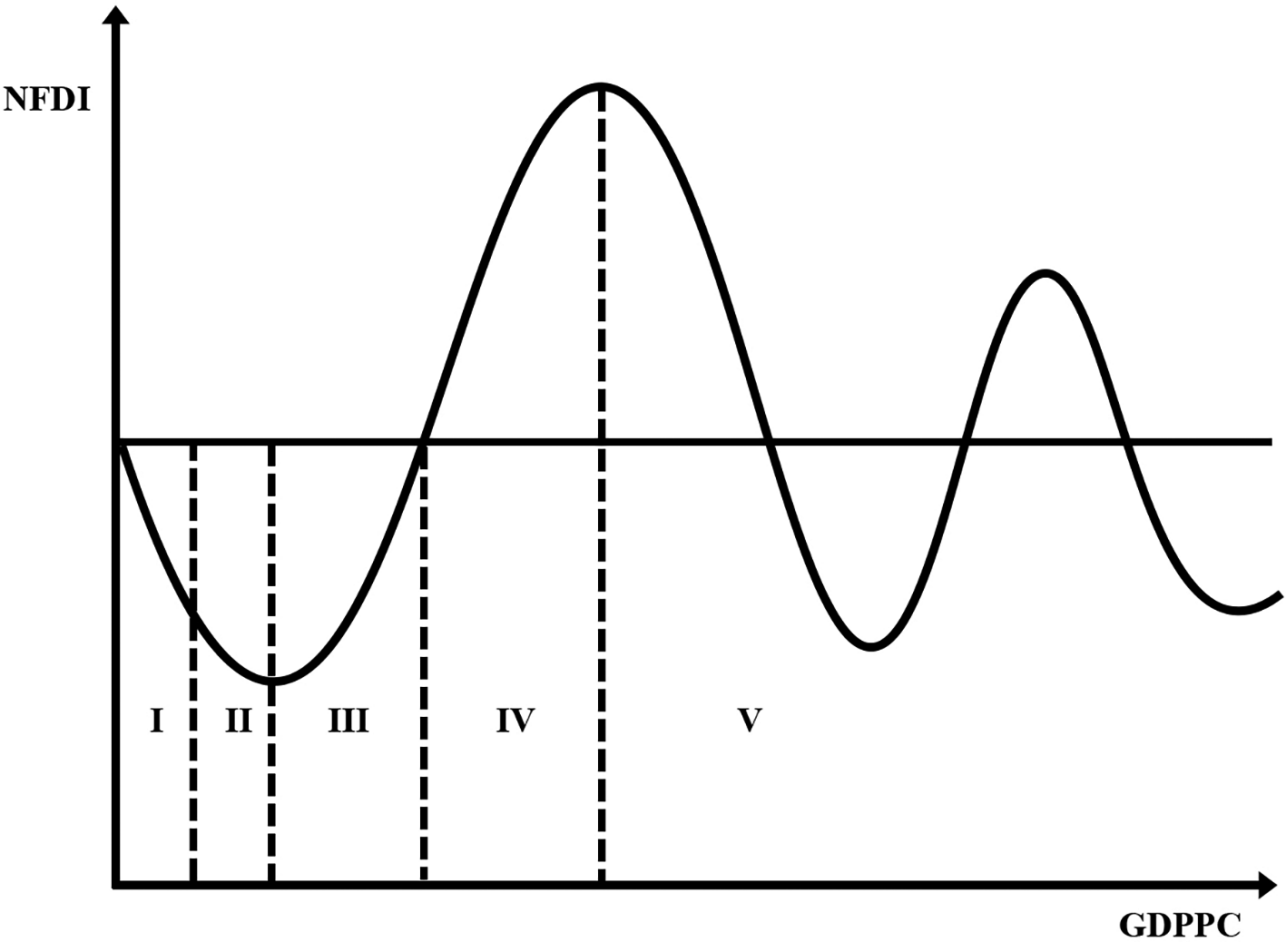
Investment development path. Note: Not drawn to scale – For illustrative purposes only.

Since the seminal work of John H. Dunning in 1979, many papers about the IDP have been published. Whilst some focused on individual countries ( Bellak, 2001; Buckley and Castro 1998; Gorynia *et al.*, 2009; Kosztowniak, 2018; Marton and McCarthy, 2007; Verma and Brennan 2011; Zhubikenov, 2022), others covered a collection of countries ( Andreff, 2003; Barry, Görg and McDowell, 2003; Borowicz, 2021; Boudier-Bensebaa, 2008; Dai, 2021; Djokoto, 2021a; Djokoto and Pomeyie, 2021; Dunning & Narula, 1996; Dunning *et al.*, 2001; Durán and Ubeda, 2001, 2005; Fonseca *et al.*, 2016; Gorynia *et al.*, 2010a, 2010b, 2012, 2019; Iacovoiu and Panait, 2014; Kuzel 2017; Ragoussis 2011; Satoglu, 2017; Trąpczyński *et al.*, 2019, 2022; Zhubikenov, 2022). Sawitri and Brennan (2022) and Serafim (2011) published some reviews. These studies have two limitations. First, notwithstanding the origin of the IDP shown in Figure 1, some studies used regression analysis without accounting for other control variables, except Djokoto (2021a). Failure to incorporate relevant control variables raises issues about the robustness and the explanatory power of the model. Second, none of these studies addressed agriculture, a sector that is critical for the survival of mankind.

Our study intended to fill these gaps by first, examining the agricultural sector of developing countries. Secondly, we considered other variables that explain net outward FDI other than the gross domestic product (GDP) per capita. Thirdly, we employed econometric estimations to support the trend line based on the scatter plot, which is the origin of the IDP. In our contribution, we found global agricultural production is in the IDP’s stages I and II. Broadly, agricultural production requires policies that would increase both outward and inward FDI. These include strong institutional support for FDI and improving the macroeconomic environment as these drive both inward and outward FDI. Promoting trade in agricultural commodities is essential in this regard.

In what follows, we review the IDP and present some empirical evidence on it. Next, we outline our empirical strategy and data. In the results and discussion section, we show the results of the selection of the appropriate model based on the information criteria, test for endogeneity and check the robustness of the IDP to estimators, charts, and control variables. The last section contains the conclusions and some policy recommendations.

## Literature review

### The theory of investment development path

According to Kumar and McLoed (1981), the theory of the IDP was propounded by John H. Dunning in 1979. This theory holds that the net outward direct investment situation of an economy is methodically correlated with its economic progress, vis-à-vis other countries. Revisions of the theory are contained in Dunning (1981a, 1986, 1988a, 1993), Narula (1993). Essentially, patterns of the relationship between GDPs per capita (GDPPC) and outward FDI less inward FDI define five idealised stages of development ( Figure 1). The FDI pattern is in turn governed by the ownership, location, and internationalisation advantages of the indigenous and foreign firms ( Dunning, 1988a, 1993, 1995, 1998).

In stage I, the place-specific merits of a country are inadequate to entice IFDI ( Dunning, 1988a, 1993, 1995, 1998). Those attributable to non-artificial assets are, however, exempted. Limited local markets, unsuitable economic arrangements, or political management strategies are evidence of the lack of place-bound assets. These can reflect insufficient infrastructure and poor capacity of the labour force. The theory notes that in stage I, outward direct investment is less probable ( Narula, 1993).

The continual little outward investment signal of a rise in inward direct investment is symptomatic of stage II ( Dunning, 1988a, 1993, 1995, 1998). Foreign firms respond to the import restriction policies adopted in response to happenings in stage I. This is reflected in foreign firms investing in import substitution industries because of either an increase in the economy's size or the citizens' purchasing power. Foreign firms take advantage of the existing level of imperceptible resources including technology, registered marks, and management skills ( Narula, 1993). The export sector would respond by increasing exports of largely non-artificial resources and prime products with some level of backward and forward linkage into labour-exhaustive little technical knowledge ( Narula, 1993). By implication, a country should have some looked-for place features to entice IFDI, contingent on its development strategy, and preference for technical capacity development of local businesses.

In stage III, IFDI decelerates whilst outward foreign direct investment (OFDI) accelerates moving the negative net OFDI towards zero. Building on the capacity and lessons from stage II, in stage III, the technological capabilities of the country are more and more directed to the production of standardised goods ( Narula, 1993). As the rise in incomes that started in stage II continues, citizens would clamour for superior attribute goods, inspired partly by the increasing keenness amid the delivering organisations ( Narula, 1993). Dunning and Narula (1996) postulate the following: 1. Relative merits in labour-exhaustive undertakings will decline. 2. Local wages will increase. 3. OFDI will be sought more and more by countries at earlier stages of the IDP. 4. The initial possession recompenses of foreign businesses also start to get worn out, as local businesses obtain the modest merits and vie with them.

In stage III, firms' ownership merits influenced by control of trademarked assets will be as those of foreign firms in the country. As domestic firms develop capacity, the role of state-engendered ownership merits is probably less important. Stage III is typical of emerging economies ( Frenken and Mbuvi, 2017).

As the OFDI continues to rise from stage III, the level rises to equal to or exceeds that of the IFDI ( Dunning, 1988a, 1993, 1995, 1998). This is stage IV. In this stage, domestic firms can now effectively vie for market and resources with foreign-owned firms in the home country and can enter foreign markets as well ( Narula, 1993). With increased human capital and technology, capital-intensive production techniques will be employed in the production of contemporary products. In the light of ownership, location and internationalisation paradigm, the location advantages will be built largely on created assets ( Dunning, 1988a, 1993, 1995, 1998). In the view of Dunning (1993), the ownership merits will be more of a ‘transaction’ than an ‘asset’. OFDI will continue to grow relative to IFDI in stage IV.

The last stage, V, is described as a home for developed countries. This stage has some features ( Dunning, 1988a, 1993, 1995, 1998). 1. There is a rising tendency for international business dealings to be internalised and become intra-multinationals rather than across borders. 2. No one country has complete domination of produced assets. 3. The multinationals’ ownership merits will depend less on their economy’s non-artificial assets’ endowment but increasingly on their capacity to obtain resources and on the capacity of businesses to form their merits competently and to feat the gains of international joint control. 4. Firms become globalised, and their countries of origin become indistinct. 5. The multinationals link geo-political gulfs and integration as such they no longer function with the interests of their countries of origin. They trade, acquire resources and process these in various countries, taking advantage of created and natural assets based on their lead best interest. 6. The ownership and geographical boundaries of the organisation become unclear as they get involved in a more and more interlocking network of trans-border supportive arrangements. 7. As the place-bound assets of countries increase in similarity, the inward and outward foreign direct investments are likely to match each other. The movements in the IFDI and OFDI would lead to unusable or fluctuating net OFDI. Essentially, the peaks and troughs of net OFDI become transient ( Narula, 1993).

It is obvious from Figure 1 that a rise in GDPPC is connected to a rise in the position of the IDP. To some extent, this supports the common practice of using GDP per capita as an indicator of development ( Abd Hakim *et al.*, 2022; Macek, 2014; McNabb, 2018; Minh Ha *et al.*, 2022; Neog and Gaur, 2020; Ranis, 2004; Radulović and Kostić, 2020; Santiago *et al.*, 2020), measured as the human development index (HDI). Recently, Djokoto (2021b) found that the IDP explains human development (HD). Further, using the GDP per capita as an indicator for IDP, Djokoto (2022) showed that GDP per capita is positively related to human development. It is worth noting that although GDP per capita is an ingredient in the construction of the HDI, it is not the only ingredient.

### Empirical evidence

The empirical literature is summarised in Table 1. Since the seminal paper of Dunning, the earliest empirical work was published in 1996 and the latest in 2022. The scope of the studies included single countries, regions, and development groups. The data structure varied from time series through cross-sectional to panel. Charts have been the main analytical tool. Others have been tables and regression analyses. There is evidence of all stages of the IDP. Whilst some empirical evidence confirmed the theoretical stages, others were inconsistent with the theory.

**Table 1.** Summary of the IDP literature.

Author(s) and date	Scope of the study	Analysis	Main results
Andreff (2003)	176 (developing and developed, 26 transition economies), 1998.	Regression	The industry distribution of gross domestic product in the home country also has an impact on the level of outward foreign direct investment. No link between OFDI and the level of technology. Developing in stage I, transition in stage II.
Barry, Görg & McDowell (2003)	Irish-US FDI relationship, 1980-1999	Regression. No controls.	Confirmation of the IDP’s idiosyncratic nature but Irish FDI outflows are disproportionately horizontal and concentrated in nontraded sectors.
Bellak (2001)	Austria, 1990-1999.	Regression. No controls.	Confirmation of the IDP’s idiosyncratic nature: the Austrian NOIP is below average and largely varies according to industry type and type of partner country.
Borowicz (2021)	Baltic states, 2004 – 2019.	Charts.	Estonia, Lithuania, and Poland: stage III. Latvia: Stages II & III.
Boudier-Bensebaa (2008)	Central and Eastern European Countries, 1991-2005.	Regression, FE. No controls	The position of the CEECs is at stage I or II of the IDP. CEECs diverged from EU15 in terms of NOIP per capita but converged in terms of GDP per capita. Less developed CEECs are converging with more developed CEECs in terms of outward investment position but not in terms of GDP per capita.
Buckley & Castro (1998)	Portugal, 1943-1966.	Regression. No controls.	- Confirmation of the IDP’s idiosyncratic nature; Beyond a country’s level of development, non-economic variables affect FDI; Replacement of the quadratic equation.
Dai (2021)	BRIC, USA, France, Germany, Japan, Singapore, Australia, Canada.	Regression. No controls.	Brazil: Stage IV. Russia: stage III & IV. China: stage II. USA, France, Germany, Japan, Singapore, Australia and Canada: Stage V.
Djokoto (2021)	Small States, 1980-2019.	Regression, RE with controls	> Half of the Small States are in stages I and II. < half are in stage III. Estonia and Malta on stage. Small States together in stage IV.
Djokoto & Pomeyie (2021)	Africa, 1991-2017.	Regression, OLS. No controls.	African countries in stage II and early stage III. Income level classifications appear to enhance the position of countries within the investment development path ahead of that based on the United Nations classification.
Dunning & Narula (1996)	Cross-section of 88 developed and developing countries, 1980 and 1992	Charts, OLS, no controls.	Confirmation of the IDP’s idiosyncratic nature; Polarization of countries into three groups.
Dunning *et al*. (2001)	Rep. of Korea, 1981-1997 and Taiwan Province of China, 1968-1997	Charts, regression. No controls.	The interface between the IDP and the trade development path.
Durán & Ubeda (2001, 2005)	85 developed and developing countries, 1997; 95 countries, 2000	Principal component analysis. Regression. No controls.	A new approach to IDP using factor analysis. Test of the power of structural variables to explain inward and outward FDI. Reformulation of the fourth stage.
Fonseca et al. (2016)	Developed countries, Portugal, 1990-2011.	Regression, FE.	Contrasts between the theory and evidence of IDP in several cases.
Gorynia et al. (2009)	Poland, 199-2006.	Charts & Tables	Manufacturing, financial intermediation, trade and repairs, transport & communications, real estate, R&D, lease: stage II. Poland: Stage II. No specific analysis of agriculture.
Gorynia et al. (2010a)	10 CEE, 1990-2008.	Charts & Tables	Some are in stage II, others in stage III.
Gorynia et al. (2010b)	Poland, Czech, Hungary, Slovakia; 1990-2006.	Charts.	Poland, Czech, Hungary, and Slovakia in stage II.
Gorynia *et al.* (2012)	Central and Eastern Europe; 1990-2008.	Charts, regression. No controls.	Countries moved from stage II into early stage III.
Gorynia et al. (2019)	Central and Eastern Europe	Regression. Controls: institutional reforms, population, year.	Most of the countries follow a quadratic relationship between the net outward investment (NOI) position. In each country's economic development, the role of institutional reforms is not in all cases accelerating the movement through the stages of the IDP.
Iacovoiu & Panait (2014)	27 EU members, 2005 and 2011.	Chart	The “narrow” version of the IDP is rather indicative. The NOFDI position is not always accurately related to the level of economic development. 2005: CEE-II, Estonia, Czech, Hungary, Slovenia-III, France, Belgium, -IV, Ireland, Denmark, V. 2011-Cyprus-III, Czech, Greece, Malta, Slovenia-IV, Belgium-V, UK, Denmark-V-IV.
Kuzel (2017)	Visegrad-Poland, Hungary, Czech, Slovakia; 1990-2013	Charts and tables	Poland is in the early stage III of the IDP.
Satoglu (2017)	Mexico, Indonesia, Nigeria, and Turkey (MINT), 1990-2013.	Regression, FE. No controls.	MINT economies are at stage II of IDP.
Trąpczyński et al. (2019)	11 CEE, 1990 – 2004.	Charts and tables.	CEE are in stage 3.
Trąpczyński et al. (2022)	Bulgaria, Albania, Romania, Georgia, 1994-2019.	Charts, Regression (OLS). No controls.	Ambiguous effect of European Union (EU) membership on IDP trajectories. Bulgaria: Early stage III; Albania, Romania and Georgia: late stage II.
Zhubikenov (2022)	Kazahkstan, 1991-2020	Charts	Stage II of IDP.

One of our previous research projects studied the IDP of Small States and segregated Small States into both developed and developing countries ( Djokoto, 2021a). The exchange rate influenced the net outward foreign direct investment per capita (NOFDIPC) for the developing, the developed and the combined sample. Inflation significantly influenced NOFDIPC for developing and developed Small States. Whilst the effect of the latter was negative, that of the former was positive. In the combined sample, the effect of inflation was neutral. Regarding human capital, the effect was significant for developing Small States and the combined sample but not for developed Small States. In the case of trade openness, the coefficient was negative and statistically significant for developed Small States but negative and statistically insignificant for developing Small States and the combined sample.

It can be observed from Table 1 that no IDP study focused on agriculture. Their assessments avoided higher-level statistical analysis. Our study goes beyond the diagrammatic illustration of the IDP to include econometric analysis with control variables. Our data covers developing countries’ agriculture.

## Data, Models and Modelling

### Study design

The quantitative research approach is employed by relying on secondary data. This has a cross-sectional dimension (countries) and a time dimension (years). Hence, panel data was used. The data were obtained from public sources.

### Model

Before the statistical analysis, we created charts of the IDP for agriculture. This involved a scatterplot of *NOFDIPC* on the vertical axis and *GDPPC* on the horizontal axis and fitted with a polynomial trend line. Although the origins of the IDP lie in the chart depiction of the nexus between NOFDIPC and GDPPC ( Figure 1) ( Dunning and Narula, 1996), statistical assessments tend to provide a more rigorous outcome ( Dai, 2021; Djokoto, 2021a; Djokoto and Pomeyie, 2021; Fonseca *et al.*, 2016; Satoglu, 2017).

The theory of IDP supposes the relationship between NOFDI and GDPPC ( Boudier Bensebaa, 2008; Djokoto and Pomeyie, 2021; Dunning, 1981a, 1986; Durán and Ubeda, 2001, 2005; Frenken and Mbuvi, 2017; Gorynia *et al.*, 2012, 2019; Iacovoiu and Panait, 2014), we specify model 1,

$$\text{NOFDIPC}=f\left(\text{GDPPC}\right)$$ (1)

*NOFDIPC* is the stock of OFDI less than the stock of IFDI divided by the total population (male and female) of a country for the corresponding year. The data on OFDI and IFDI for agriculture was reported as flows and not stocks. Consequently, the first observation for every country was considered the initial flow. The subsequent flows were added consecutively to build the stock. *GDPPC* is nominal GDP divided by the total population. We follow Dunning (1981a, 1986, 1988a, 1993) in this regard.

However, other factors could explain *NOFDIPC* other than *GDPPC* ( Andreff, 2003; Djokoto, 2021a; Durán and Ubeda, 2001, 2005; Frenken and Mbuvi, 2017). These can be discussed from two perspectives: the host country and the home country ( Paul, 2014). We focus on the home country determinants based on the data employed. In a review of IDP studies, Sawitri and Brennan (2022) identified factors including international trade, exchange rate, human capital and inflation as other determinants of OFDI.

Hence,

$$\text{NOFDIPC}=f\left(\text{GDPPC},\text{AGTO},\text{EXRATE},\mathrm{HC},\text{INFLA}\right)$$ (2)

Where the other variables are controls, namely trade openness ( *AGTO*), the exchange rate ( *EXRATE*), human capital ( *HC*) and inflation ( *INFLA*). *AGTO* is defined as the sum of exports and imports to the ratio of the gross domestic product for the agricultural sector. Both inward and outward FDI engenders trade. Accessing new markets by multinationals (MNEs) could start with exports of finished products to the to-be host country. Whilst in the host country, the AM could export intermediate finish products to the home country as well as other countries. Where resources are the attraction for the AM, trade could involve imports from the to-be host country. Thus, trade influences both outward FDI and inward FDI ( Buckley *et al.*, 2007; Djokoto, 2021a; Frenken and Mbuvi, 2017; Tolentino, 2008). *EXRATE* is the official exchange rate captured as the annual average of the local currency per US dollar. The increase in the value of the local currency will cause a decline in local currency resources to invest abroad ( Buckley *et al.* 2007; Paul, 2014) which enhances the level of outward FDI. Also, the increase in value of the local currency, makes products and services more expensive. This decreases the attractiveness of exports relative to FDI; hence, positively influences FDI going out. Thus, the effect of the local currency’s value may harm FDI going out ( Bhasin and Jain 2013; Djokoto, 2021a, Paul, 2014).

*HC* was captured as the secondary school enrolment as a percentage of gross enrolment following Djokoto (2022). A skilled labour force is an ownership advantage that firms must possess to engage in outward FDI and to support inward FDI. Thus, human capital influences both outward and inward FDI ( Djokoto, 2021a; Stoian, 2013; Tolentino, 2008).

*INFLA* was defined as the annual growth rate of the consumer price index following Djokoto (2023), Amal and Tomio 2012; Djokoto, 2021a; Paul, 2014 and World Bank (2023a). In the presence of low macroeconomic stability, businesses will probably seek stable economic environments outside the home country. Proxying economic stability by inflation, a less volatile or more volatile inflation rate points to a positive business environment, that encourages a firm's outward relocation ( Amal and Tomio 2012; Djokoto, 2021a; Paul, 2014). We specified equation 2, thus:

$${\text{NOFDIPC}}_{i,t}=\alpha_{0}+\alpha_{1}{\text{GDPPC}}_{\mathrm{it}}+\alpha_{2}{\text{GDPPC}}_{\mathrm{it}}^{2}+\alpha_{3}{\text{GDPPC}}_{\mathrm{it}}^{3}+\alpha_{4}{\text{AGTO}}_{\mathrm{it}}+\alpha_{5}{\text{EXRATE}}_{i,t}+\alpha_{6}{\mathrm{HC}}_{\mathrm{it}}+\alpha_{7}{\text{INFLA}}_{\mathrm{it}}+\epsilon_{\mathrm{it}}$$ (3)

Equation 3 was estimated for the appropriate powers of the *GDPPC* that were established econometrically.

### Data

The data consists of a panel of 55 developing countries in Table 2 from 1991 to 2021. The data include a total of 885 observations. Data to construct *NOFDIPC* and *GDPPC* were obtained from the Food and Agriculture Organization Corporate Statistical Database (FAOSTAT) (2023a, b) whilst data for *EXRATE*, *HC* and *INFLA* were obtained from the World Development Index of the World Bank (2023a, b). The countries included in the data were based on the availability of data from the sources. Further, to ensure consistency, all data was extracted from the United Nations data system.

**Table 2.** List of developing countries in the data.

Country	Country	Country	Country
Algeria	El Salvador	Mexico	Saudi Arabia
Bangladesh	Ethiopia	Morocco	Solomon Islands
Belize	Ghana	Mozambique	Syrian
Bhutan	India	Myanmar	Thailand
Bolivia	Indonesia	Nepal	Tunisia
Brazil	Iran	Nicaragua	Türkiye
Cambodia	Jamaica	Nigeria	Uganda
China, mainland	Jordan	Pakistan	United Arab Emirates
Colombia	Kenya	Panama	United Republic of Tanzania
Costa Rica	Laos	Papua New Guinea	Uruguay
Côte d'Ivoire	Madagascar	Paraguay	Vanuatu
Djibouti	Malawi	Peru	Viet Nam
Ecuador	Malaysia	Philippines	Zambia
Egypt	Mauritius	Rwanda	

### Estimation procedure

First, we established the polynomial order of the *GDPPC* using the information criteria ( Akaike, 1974; Schwarz, 1978). Second, we expressly tested for the existence of endogeneity between our key variables, *NOFDIPC* and *GDPPC.* Thirdly, we estimated equation 3 using panel fixed effects (FE) and random effects (RE) estimators, selected the appropriate specification based on the Hausman test ( Hausman, 1978) and tested for violations of the classical regression; serial correlation ( Wooldridge, 2002) and heteroscedasticity using the modified Wald test for heteroskedasticity ( Greene, 2000). The third step was applied to each model during the robustness check of the estimates of the *GDPPC, GDPPC ^2^* and *GDPPC ^3^* to the control variables.

## Results and discussions

### Descriptive Statistics

The minimum *NOFDIPC* is -180.1437 (Uruguay, 2008) with a maximum of 32.5600 (Malaysia, 2011) ( Table 3). The mean of -4.4210 is close to that of Mozambique in 2008. Based on the standard deviation of 17.2928, the variance is more than the mean suggesting overdispersion of the data. Similar overdispersion can be observed with *GDPPC* ^2^. Except for *GDPPC* and *HC* for which the standard deviation is less than the respective means, for all other control variables, the standard deviation exceeds the mean.

**Table 3.** Descriptive statistics.

Variable	Observations	Mean	Standard deviation	Minimum	Maximum
*NOFDIPC*	885	-4.4210	17.2928	-180.1437	32.5600
*GDPPC*	885	312.2789	207.6065	27.2041	1439.9630
*GDPPC2*	885	1.40e+05	2.18e+05	740.0643	2.07e+06
*GDPPC3*	885	8.60e+07	2.40e+08	20132.8	2.99e+09
*AGTO*	885	260.2053	384.5166	3.803688	3411.748
*EXRATE*	885	1513.047	3713.032	.0026086	25941.66
*HC*	885	71.21255	28.98276	5.2834	157.7033
*INFLA*	885	17.67854	251.8703	-8.484249	7481.664

### Determining the order of the polynomial

**Figure 2.**
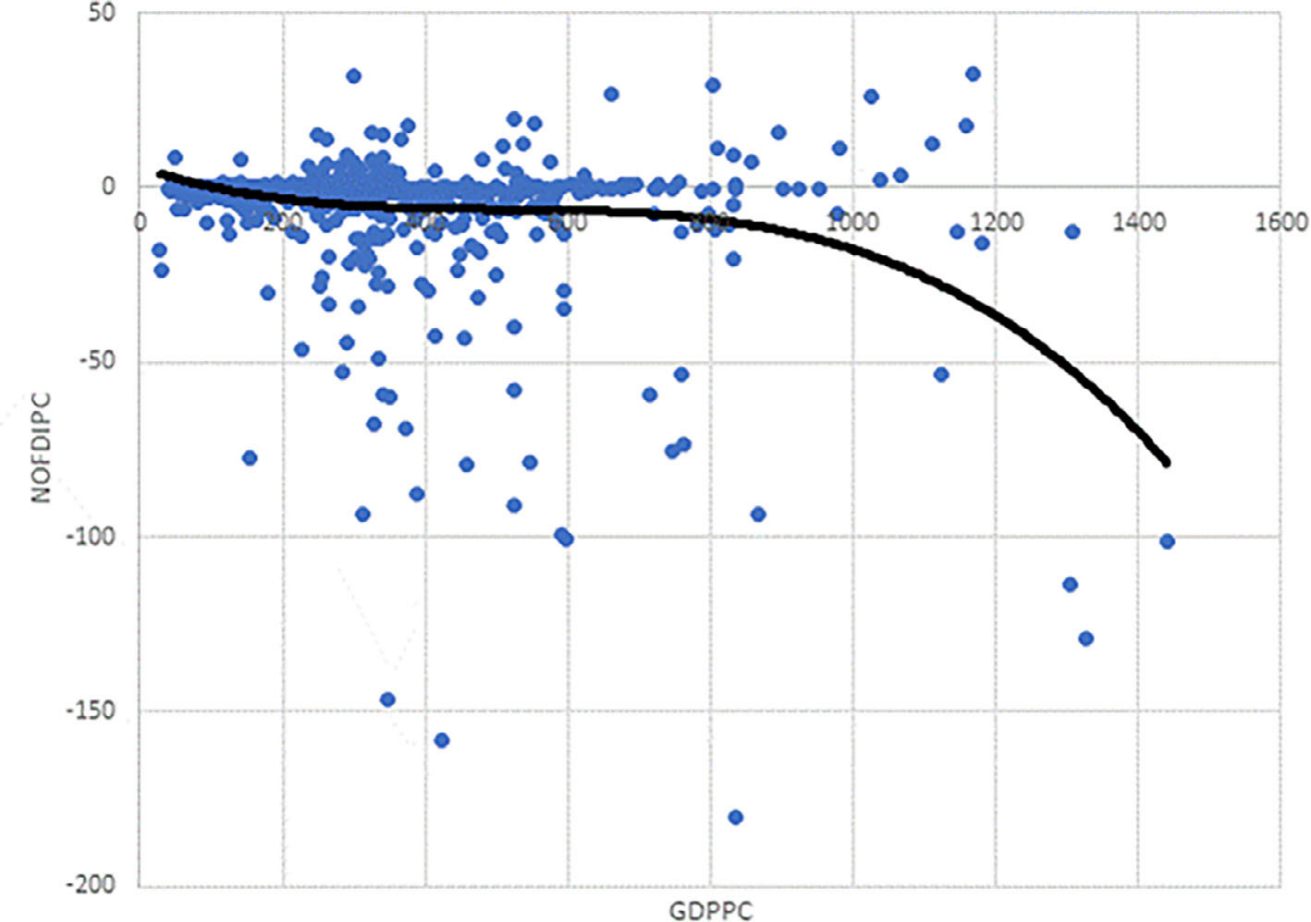
Chart of investment development path for developing countries' agriculture.

As the observation in Figure 2 is based on the scatter plot of *NOFDIPC* and *GDPPC*, it may well be that incorporating other control variables could change this. Thus, we estimated models 1 – 3 to test this. The AIC and BIC for model 3 are the lowest ( Table 4). This suggests that model 3, with the cubic functional form, a polynomial of order 4 is appropriate for the curve based on the estimations. This conforms to the cubic curve shown as the trend line in Figure 2. This is in line with our specification of equation 3 to be modelled.

**Table 4.** Selection of order of the polynomial.

	(1)	(2)	(3)
*VARIABLES*	*NOFDIPC*	*NOFDIPC*	*NOFDIPC*
*GDPPC*	-0.00007 (0.00146)	0.00330 (0.00586)	-0.03953 *** (0.01267)
*GDPPC2*		-0.00000 (0.00000)	0.00004 *** (0.00001)
*GDPPC3*			-0.00000 *** (0.00000)
*INFLA*	0.00191 (0.01163)	0.00208 (0.01164)	0.00070 (0.01150)
*EXRATE*	0.00103 (0.00378)	0.00082 (0.00380)	0.00254 (0.00378)
*HC*	0.01975 (0.02991)	0.01416 (0.03137)	0.03043 (0.03126)
*AGTO*	0.00152 ** (0.00072)	0.00148 ** (0.00072)	0.00180 ** (0.00072)
Constant	-4.39275 (2.94576)	-5.08533 (3.16886)	3.15229 (3.80602)
Model diagnostics			
Observations	519	519	519
R-squared	0.01037	0.01106	0.03823
AIC	7500	7491	7477
BIC	7529	7524	7515

Notes: 1. Standard errors in parenthesis. 2.

*** p<0.01.

** p<0.05.

* p<0.1. 3.

### Test for endogeneity

In an earlier study with other colleagues, we found that agricultural FDI explained agricultural GDP ( Djokoto *et al.*, 2022). Other studies made similar findings (( Djokoto, 2013; Gunasekera *et al.*, 2015) whilst the reverse is also true ( Djokoto, 2012; Kassem and Awad, 2019; Lv *et al.*, 2010), hence there can be an endogeneity problem. Rather than anticipate and model accordingly, we proceeded to test for endogeneity ( Table 5). First, we modelled *GDPPC* as the dependent variable with all others as exogenous variables. We predicted the errors *GDPPC_ue* and did the same for *GDPPC ^2^* and obtained *GDPPC ^2^_ue* as well as *GDPPC ^3^* and obtained *GDPPC ^3^_ue.* In Model 7, the predicted terms were introduced as additional explanatory variables. We then tested the significance of the *GDPPC_ue*, *GDPPC ^2^_ue* and *GDPPC ^2^_ue* with a chi-square test. Failing to reject the null hypothesis suggests that the error terms are not correlated with the *NOFDIPC*, hence the suspicion of endogeneity is not borne out by the data. Following the absence of endogeneity, equation 3 was estimated with FE and RE estimators. The Hausman test showed that the hull hypothesis that the differences between the matched coefficients are not systematic could not be rejected. Consequently, the RE estimation was preferred to fixed effects. The errors were found to be serially correlated and non-constant (heteroscedastic). We addressed this with robust standard errors. The serial correlation was resolved by introducing a first lag of *NOFDIPC*, *d.NOFDIPC* as appropriate.

**Table 5.** Test for endogeneity.

	(4)	(5)	(6)	(7)
*VARIABLES*	*GDPPC*	*GDPPC2*	*GDPPC3*	*NOFDIPC*
*GDPPC*				0.0213 (0.1864)
*GDPPC_ue*				-0.0609 (0.1876)
*GDPPC2*				-0.0000 (0.0001)
*GDPPC ^2^_ue*				0.0001 (0.0001)
*GDPPC3*				0.0000 (0.0000)
*GDPPC ^3^_ue*				-0.0000 (0.0000)
*AGTO*	0.1805 *** (0.0120)	328.8868 *** (37.4777)	776,480.8601 *** (134,697.2278)	-0.0015 (0.0077)
*INFLA*	-0.0370 (0.0720)	96.6471 (224.8143)	433,938.3555 (822,108.9388)	
*EXRATE*	0.0422 (0.0278)	98.8198 (86.8459)	323,267.9576 (317,452.2456)	
*HC*	-2.3266 *** (0.5950)	-4,374.0303 ** (1,852.0479)	-7.5614e+06 (6667281.2236)	
Constant	538.4363 *** (110.6878)	499,744.8123 (320,430.3540)	5.2201e+08 (9.4146e+08)	-1.3148 (43.2050)
Model diagnostics				
Observations	519	519	519	519
Countries	26	26	26	26
Chi-square test				
*GDPPC_ue*				0.01
*GDPPC ^2^_ue*				0.02
*GDPPC ^3^_ue*				0.00

Notes: 1. Standard errors in parenthesis. 2.

AGTO and GGI were omitted because of collinearity.

*** p<0.01.

** p<0.05.

* p<0.1. 3.

### Robustness

The estimates of *GDPPC*, *GDPPC ^2^* and *GDPPC ^3^* in 8 are consistent with those of models 9 – 13 ( Table 6). The estimates of *AGTO, EXRATE, HC* and *INFLA* are also consistent across models 9 – 12 and model 13.

**Table 6.** Random effects estimations of IDP and robustness of estimates to control variables.

	(8)	(9)	(10)	(11)	(12)	(13)
VARIABLES	*NOFDIPC*	*NOFDIPC*	*NOFDIPC*	*NOFDIPC*	*NOFDIPC*	*NOFDIPC*
*D.NOFDIPC*				0.5188 *** (0.0270)	0.5189 *** (0.0269)	
*GDPPC*	-0.0613 * (0.0337)	-0.0631 * (0.0329)	-0.0587 * (0.0326)	-0.0822 * (0.0431)	-0.0798 ** (0.0402)	-0.0629 * (0.0348)
*GDPPC2*	0.0001 * (0.0001)	0.0001 * (0.0001)	0.0001 (0.0001)	0.0001 * (0.0001)	0.0001 * (0.0001)	0.0001 (0.0001)
*GDPPC3*	-5.16e-08 (3.25e-08)	-5.16e-08 * (3.11e-08)	-5.05e-08 (3.22e-08)	-7.45e-08 * (3.93e-08)	-7.38e-08 * (3.84e-08)	-5.12e-08 (3.16e-08)
*AGTO*		0.0063 * (0.0034)				0.0061 * (0.0032)
*EXRATE*			-0.0002 (0.0004)			-0.0002 (0.0004)
*HC*				0.0165 (0.0360)		0.0200 (0.0434)
*INFLA*					0.0077 (0.0161)	-0.0002 (0.0002)
Constant	2.3211 (3.7669)	1.6881 (3.5617)	2.1155 (3.6227)	4.0253 (3.7165)	4.7073 (4.2542)	0.5120 (3.1361)
Model diagnostics						
Observations	885	885	885	783	783	885
Countries	55	55	55	55	55	55

Notes: 1. Robust standard errors in parenthesis. 2.

* p<0.10.

** p<0.05.

*** p<0.01.

### Discussion of the IDP for agriculture

We focus on model 13 for discussions. The negative and statistically significant coefficient of *GDDPC* confirms the curve declines initially as in Figure 2. The statistically insignificant coefficients of *GDPPC ^2^* and *GDPPC ^3^* suggest points of inflexion. The positive and negative signs respectively show the rise and fall before and after the points of inflexion. The estimates correctly depict Figure 2 notwithstanding the introduction of the control variables. It is informative to note that notwithstanding the cubic function in Figure 2, confirmed by the model selection in Table 2 and estimates in Table 5, the curve remains below the *GDPPC* axis. Indeed, the curve does not show any significant rise after its first decline. Juxtaposing Figure 2 and Figure 1, developing agriculture is in stages I and II of the IDP. The stage I and II found is in line with the theory of the IDP proposed by Dunning (1981a, 1986, 1988a, 1993) and Narula (1993). This is consistent with the finding for the total economy studies for developing countries ( Andreff, 2003; Satoglu, 2017) and other regions ( Boudier-Bensebaa, 2008; Borowicz, 2021; Dai, 2021; Gorynia *et al.*, 2009, 2010a, b, 2012; Zhubikenov, 2022). However, total economy studies of other regions found IDP stages other than I and II ( Djokoto, 2021a; Iacovoiu and Panait, 2014; Kuzel, 2017; Trąpczyński *et al.*, 2019).

The statistical significance of the *GDPPC* is in line with the evidence that the size of the economy determines FDI in agriculture ( Djokoto, 2012; Djokoto, 2021c; Husmann and Kubik, 2019; Rashid and Razak, 2017). It also confirms the outcome of early studies of Dunning (1981a, 1986, 1988a, 1993) and Narula (1993). The statistical significance of the *GDPPC* also confirms the existence of the IDP. This should be viewed within the framework of the evidence that GDPPC, IDP and HD are positively correlated ( Djokoto, 2021a, b, c, 2022; Djokoto and Pomeyie, 2021).

The continual low or negligible OFDI and rise in IFDI are observable. The outcome means that the location-specific advantages of developing countries are inadequate to attract IFDI. These can be attributable to inadequate infrastructure and poor capacity of the labour force. Foreign firms may be interested in investing in import substitution industries because of either an increase in the economy's size or the citizens' purchasing power. The weakness of the developing countries encourages foreign firms to take advantage of the existing level of imperceptible resources including know-how, registered marks, and managerial expertise. Foreign firms may also invest in export sectors increasing exports of largely non-artificial resources and prime commodities with some level of backward and forward linkage integration into labour- exhaustive low know-how.

### Discussion of control variables

The positive and statistically significant coefficient of *AGTO* implies that trade enhances *NOFDI.* Aside from accessing new markets with new products at the start of internationalisation, home, and foreign affiliates engage in the trade of raw materials and finished and semi-finished products. Where divestments occur, the markets could be filled with products from foreign affiliates of the home country. In all these, the constituents of trade openness, imports and exports take place, hence, this is a positive sign. Djokoto (2021a) found a negative sign of the estimates of trade that were statistically insignificant for both developing and the combined Small States except for developed Small States. Djokoto (2021a) did not provide reasons for the results on the trade variable.

The negative sign of the coefficient of *EXRATE* is consistent with that of Djokoto (2021a), there is a departure regarding the statistical significance. The coefficient of *INFLA* is also negative but statistically insignificant. Our finding is contrary to that of Djokoto (2021a) who found positive and statistically significant coefficients for developing Small States as well as the combined sample, but negative and statistically significant coefficients for developed Small States. As in the case of the other control variables, Djokoto (2021a) did not provide reasons for the significant coefficients.

Regarding the control variables, in summary, agricultural trade openness enhances agricultural net foreign direct investment whilst the exchange rate has the opposite effect. Inflation does not affect agricultural net foreign direct investment.

## Conclusions and recommendations

We employed data from 1991 to 2021 for 55 developing countries to empirically investigate the IDP for agriculture in developing countries. Our analytical approach indirectly accounts for interactions among countries regarding cross-border resource transfers. Aside from providing knowledge on testing the IDP by inferential statistics, the information would be relevant for policy. Further, the stage of the IDP reflects the cross-border attractiveness of agriculture and tortuously of agricultural businesses going abroad from the agricultural sector and gives guideposts for apt economic strategy.

We found agricultural production in developing countries in IDP’s stages I and II. Broadly, agricultural production requires policies that would increase both OFDI and IFDI. Agricultural multinationals in developing countries must develop capacity by learning from foreign multinationals. This would enable them to go abroad. Such a move would lead to an increase in OFDI. As this would have resulted from increased GDP per capita, it will lead to movement from the existing stage to higher ones. At the macroeconomic level, the government must support the building of strong institutions for FDI and improving the macroeconomic environment as these drive both OFDI and IFDI. Promoting trade in agricultural commodities is essential in this regard.

Our study is limited to developing countries. Further studies can also explore the role of the IDP in transition and or developed countries.

## Data Availability

Data for foreign direct investment and gross domestic product for agriculture were obtained from the Food and Agricultural Organisation of the United Nations dataset for various countries. These are available from the FAOSTAT ( https://www.fao.org/faostat/en/#data/FDI). At the link select developing countries from the ‘COUNTRIES’ pane. Next, select Value US$ from the ‘ELEMENTS’ pane. In the ‘ITEMS’ pane, select FDI inflows to Agriculture, Forestry and Fishing and FDI inflows to Agriculture, Forestry and Fishing. In the ‘YEARS’ pane, select 1991 to 2021. Regarding agricultural GDP, at https://www.fao.org/faostat/en/#data/MK, follow the same steps for ‘COUNTRIES’ and ‘YEARS’. For the other panes, select Value US$ and Value added (Agriculture, Forestry and Fishing) for ‘ELEMENTS’ and ‘YEARS’ respectively. Regarding the other data, these are obtainable at the Databank of the World Bank ( https://databank.worldbank.org/source/world-development-indicators). At the country tab at the link, select developing countries. Select the Official exchange rate (LCU per US$, period average), Inflation, consumer prices (annual %) and School enrolment, secondary (% gross) at the series tab. Finally, at the Time tab, select 1991, 1992, 1993 …. 2021. At the top right of the page click ‘download’. Please note that the FAOSTAT and the World Bank databases are publicly available.

## References

[ref1] Abd Hakim T Karia AA David J : Impact of direct and indirect taxes on economic development: A comparison between developed and developing countries. *Cogent Economics & Finance.* 2022;10(1):2141423. 10.1080/23322039.2022.2141423

[ref2] Akaike H : A new look at the statistical model identification. *IEEE Trans. Autom. Control.* 1974;19(6):716–723. 10.1109/TAC.1974.1100705

[ref3] Amal M Tomio BT : Determinants of Brazilian Outward Foreign Direct Investment (OFDI): A Host Country Perspective. *Presented at Encontro da ANPAD 22-26 September, Rio de Janeiro.* 2012.

[ref102] Andreff V : The newly emerging TNCs from economies in transition: a comparison with Third World outward FDI. *Transnational corporations.* 2003;12(2):73–118.

[ref4] Barry F Görg H McDowell A : Outward FDI and the investment development path of a late-industrializing economy: evidence from Ireland. *Reg. Stud.* 2003;37(4):341–349. 10.1080/0034340032000074389

[ref5] Bellak C : The Austrian investment development path. *Transl. Corp.* 2001;10(2):107–134.

[ref6] Bhasin N Jain V : Home Country Determinants of Outward FDI: A Study of Select Asian Economies. SSRN 2206739. 2013.

[ref7] Borowicz A : Evaluation of the Investment Development Path concept in selected Baltic Sea Region states: Where are we? *Rocznik Instytutu Europy Środkowo-Wschodniej.* 2021;19(3):77–100. 10.36874/RIESW.2021.3.4

[ref8] Boudier-Bensebaa F : FDI-assisted development in the light of the investment development path paradigm: Evidence from Central and Eastern European countries. *Transl. Corp.* 2008;17(1):37.

[ref9] Buckley PJ Castro FB : The investment development path: the case of Portugal. *Transl. Corp.* 1998;7:1–16.

[ref11] Buckley PJ Clegg JJJ Cross AR : The determinants of Chinese outward foreign direct investment. *J. Int. Bus. Stud.* 2007;38(4):499–518. 10.1057/palgrave.jibs.8400277

[ref12] Chen Q Guo P : Outward foreign direct investment in agriculture by Chinese companies: land grabbing or win–win? *Economic and Political Studies.* 2017;5(4):404–420. 10.1080/20954816.2017.1384607

[ref13] Dai K : Investment development path: the applicability of measurement criteria and further development. *E3S Web of Conferences.* 2021; Vol.275: p.01023. EDP Sciences.

[ref14] Djokoto JG : An investigation of the determinants of inward foreign direct investment flow into Ghana’s agricultural Sector. *Pentvars Business Journal.* 2012;6(1):19–37.

[ref15] Djokoto JG : Openness and agricultural performance in Ghana. *Journal of Science and Technology (Ghana).* 2013;33(2):24–36. 10.4314/just.v33i2.3

[ref16] Djokoto JG : The investment development path theory and small states. *Research in Globalization.* 2021a;3:100048. 10.1016/j.resglo.2021.100048

[ref17] Djokoto J : Do the stages of the investment development path explain the stages of human development? *Academia Letters.* 2021b. Article 2948. 10.20935/AL2948

[ref18] Djokoto JG : Drivers of agricultural foreign divestment. *Studies in Agricultural Economics.* 2021c;123(1):43–51.

[ref19] Djokoto JG Pomeyie P : Level of income and the investment development path theory: Evidence from Africa. *SAGE Open.* 2021;11(4):215824402110613. 10.1177/21582440211061334

[ref20] Djokoto JG : The investment development path and human development: Is there a nexus? *Research in Globalization.* 2022;4:100079. 10.1016/j.resglo.2021.100079

[ref21] Djokoto JG Gidiglo FK Srofenyoh FY : Agricultural development in the presence of foreign divestment: Policy options. *Journal of Agriculture and Food Research.* 2022;7:100250. 10.1016/j.jafr.2021.100250

[ref22] Djokoto JG : Is foreign aid effective in the food manufacturing sector?. *Journal of Agriculture and Food Research.* 2023;12:100617. 10.1016/j.jafr.2023.100617

[ref24] Dunning JH : Explaining the international direct investment position of countries: Towards a dynamic or developmental approach. *Weltwirtschaftliches Arch.* 1981a;117(30):30–64. 10.1007/BF02696577

[ref25] Dunning JH : *International Production and the Multinational Enterprise.* London: George Allen & Unwin;1981b.

[ref26] Dunning JH : The investment development cycle revisited. *World Economic Archives.* 1986;122(4):667–676. 10.1007/BF02707854

[ref27] Dunning JH : *Explaining International Production.* London: Unwin Hyman;1988.

[ref30] Dunning JH : *Multinational Enterprises and the Global Economy.* Wokingham, England, and Reading, MA: Addison-Wesley;1993.

[ref32] Dunning JH : Reappraising the eclectic paradigm in an age of alliance capitalism. *J. Int. Bus. Stud.* 1995;26(3):461–491. 10.1057/palgrave.jibs.8490183

[ref33] Dunning JH : Location and the multinational enterprise: a neglected factor? *J. Int. Bus. Stud.* 1998;29(1):45–66. 10.1057/palgrave.jibs.8490024

[ref103] Dunning JH Kim CS Lin JD : Incorporating trade into the investment development path: A case study of Korea and Taiwan. *Oxford development studies.* 2001;29(2):145–154. 10.1080/13600810123926

[ref35] Dunning JH Narula R : The investment development path revisited. *Foreign Direct Investment and Governments: Catalysts for Economic Restructuring.* 1996;1–41.

[ref36] Dunning JH Narula R : The Investment Development Path Revisited. Dunning JH , editors. *Theories and Paradigms of International Business Activity. The Selected Essays of John H. Dunning.* Cheltenham, UK and Northhampton, MA: Edward Elgar;2002;1: pp.138–172.

[ref37] Durán JJ Ubeda F : The investment development path: a new empirical approach and some theoretical issues. *Transl. Corp.* 2001;10(2):1–34.

[ref38] Durán JJ Ubeda F : The investment development path of newly developed countries. *Int. J. Econ. Bus.* 2005;12(1):123–137. 10.1080/1357151042000323076

[ref39] FAOSTAT: *Foreign Direct Investment.* Food and Agricultural Organisation of the United Nations;2023a. Reference Source

[ref40] FAOSTAT: *Foreign Direct Investment.* Food and Agricultural Organisation of the United Nations;2023b. Reference Source

[ref41] Fonseca M Mendonça A Passos J : The paradigm of the investment development path: Does it holds for Portugal? Evidence for the period 1990-2011. Instituto Superior de Economia e Gestão – CEsA/CSG Documentos de Trabalho n° 139/2016. 2016.

[ref42] Frenken JH Mbuvi D : Country risk, FDI flows and convergence trends in the context of the Investment Development Path. *UNU-MERIT Working Papers.* 2017;5:1–17.

[ref43] Gorynia M Nowak J Wolniak R : Poland's investment development path: In search of a synthesis. *International Journal of Economic Policy in Emerging Economies.* 2009;2(2):153–174. 10.1504/IJEPEE.2009.027635

[ref44] Gorynia M Nowak J Wolniak R : Investment development paths of Central European countries: a comparative analysis. *Argumenta Oeconomica.* 2010a;24(1):65–87.

[ref45] Gorynia M Nowak J Wolniak R : Foreign direct investment of Central and Eastern European countries and the investment development path revisited. *Eastern Journal of European Studies.* 2010b;1(2):21.

[ref46] Gorynia M Nowak J Tarka P : Foreign direct investment in new EU member states from Central and Eastern Europe: an investment development path perspective. *Internationalization of emerging economies and firms.* 2012;64–86. 10.1057/9780230363663_4

[ref48] Gorynia M Nowak J Trąpczyński P : Friend or foe? On the role of institutional reforms in the investment development path of Central and East European economies. *Int. Bus. Rev.* 2019;28(3):575–587. 10.1016/j.ibusrev.2018.12.003

[ref49] Greene W : *Econometric Analysis.* Upper Saddle River, NJ: Prentice-Hall;2000.

[ref50] Gunasekera D Cai Y Newth D : Effects of foreign direct investment in African agriculture. *China Agricultural Economic Review.* 2015;7(2):167–184. 10.1108/CAER-08-2014-0080

[ref51] Hausman JA : Specification tests in econometrics. *Econometrica.* 1978;46:1251–1271. 10.2307/1913827

[ref52] Husmann C Kubik Z : *Foreign direct investment in the African food and agriculture sector: trends, determinants and impacts* (No. 1546-2019-1582). 2019.

[ref53] Iacovoiu VB Panait M : The limitation of investment development path theory. European Union Case. *Petroleum-Gas University of Ploiesti Bulletin, Technical Series.* 2014;66(4):33–40.

[ref54] Ju P Anser MK Osabohien R : Trade Openness, Foreign Direct Investment and Sustainable Agriculture in Africa Otwartość handlu, bezpośrednie inwestycje zagraniczne i zrównoważone rolnictwo w Afryce. *Problemy Ekorozwoju.* 2022;17(1):246–255. 10.35784/pe.2022.1.22

[ref55] Kassem AZE Awad MM : Determinants of foreign direct investment in Egypt’s agriculture. *Assiut Journal of Agricultural Sciences.* 2019;50(4):13–14.

[ref101] Kosztowniak A : Changes of inward and outward FDI stocks in Poland and the stage of the investment development path. *Journal of Management and Financial Sciences* .35:41–60. 10.33119/JMFS.2018.35.3

[ref57] Kumar K Mcleod M : *Multinationals from Developing Countries.* Lexington, MA: D. C. Heath;1981.

[ref58] Kuzel M : The investment development path: Evidence from Poland and other countries of the Visegrad group. *Journal of East-West Business.* 2017;23(1):1–40. 10.1080/10669868.2016.1180659

[ref59] Lv L Wen S Xiong Q : Determinants and performance index of foreign direct investment in China's agriculture. *China Agricultural Economic Review.* 2010;2(1):36–48. 10.1108/17561371011017487

[ref60] Macek R : The impact of taxation on economic growth: Case study of OECD countries. *Review of economic perspectives.* 2014;14(4):309–328. 10.1515/revecp-2015-0002

[ref61] McNabb K : Tax structures and economic growth: New evidence from the government revenue dataset. *J. Int. Dev.* 2018;30(2):173–205. 10.1002/jid.3345

[ref62] Minh Ha N Tan Minh P Binh QMQ : The determinants of tax revenue: A study of Southeast Asia. *Cogent Economics & Finance.* 2022;10(1):2026660. 10.1080/23322039.2022.2026660

[ref64] Marton K McCarthy C : Is China on the investment development path?. *Journal of Asia Business Studies.* 2007;1:1–9. 10.1108/15587890780001290

[ref104] Narula R :1993; *Technology, international business and Porter's “Diamond”: Synthesizing a dynamic competitive development model* . MIR: Management International Review:85–107.

[ref65] Neog Y Gaur AK : Tax structure and economic growth: A study of selected Indian states. *Journal of Economic Structures.* 2020;9:1–12. 10.1186/s40008-020-00215-3

[ref67] Paul T : *Analysis of the Investment Development Path in the Central and Eastern European Countries: Can they move further?* Charles University in Prague;2014.

[ref69] Ragoussis A : The investment development path in space. *Rev. World Econ.* 2011;147(3):527–541. 10.1007/s10290-011-0089-7

[ref70] Ranis G : *Human Development and Economic Growth. Economic Growth Center, Center Discussion Paper. 887.* New Haven, CT: Yale University;2004.

[ref71] Rashid IMA Razak NAA : Economic determinants of Foreign Direct Investment (FDI) in agriculture sector based on selected developing OIC countries: an empirical study on the provincial panel data by using Stata, 2003-2012. *Jurnal Intelek.* 2017;12(1):6–11.

[ref72] Radulović M Kostić M : Globalization and economic growth of Eurozone economies. Zbornik Radova Ekonomskog Fakulteta u Rijeci: Časopis Za Ekonomsku Teoriju i Praksu/ *Proceedings of Rijeka Faculty of Economics.* *J. Econ. Bus.* 2020;38(1). 10.18045/zbefri.2020.1.183

[ref73] Santiago R Fuinhas JA Marques AC : The impact of globalization and economic freedom on economic growth: the case of the Latin America and Caribbean countries. *Econ. Chang. Restruct.* 2020;53(1):61–85. 10.1007/s10644-018-9239-4

[ref74] Satoglu EB : Emerging through foreign investment: investment development path estimation of MINT economies. *Advances in Economics & Business.* 2017;5(5):256–264. 10.13189/aeb.2017.050503

[ref75] Sawitri KA Brennan L : The investment development path literature: a review and research agenda. *Management Review Quarterly.* 2022;1–48. 10.1007/s11301-022-00287-4

[ref76] Schwarz G : Estimating the dimension of a model. *Ann. Stat.* 1978;6:461–464. 10.1214/aos/1176344136

[ref77] Serafim TO : The investment development path–literature review. *Annals of the “Ovidius” University.* *Economic Sciences Series.* 2011;11(1):1–5.

[ref78] Stoian C : Extending Dunning's investment development path: The role of home country institutional determinants in explaining outward foreign direct investment. *Int. Bus. Rev.* 2013;22(3):615–637. 10.1016/j.ibusrev.2012.09.003

[ref79] Tian J : Does agricultural official development assistance facilitate foreign direct investment in agriculture: Evidence from 63 developing countries. *J. Agric. Econ.* 2023;74:702–718. 10.1111/1477-9552.12527

[ref106] Tolentino PE : The determinants of the outward foreign direct investment of China and India: Whither the home country? *Working Paper Series,* #2008-049.2008.

[ref80] Trąpczyński P Gorynia M Nowak J : EU countries from central and Eastern Europe, and the investment development path model: a new assessment. *Argumenta Oeconomica.* 2019;2(43):385–406. 10.15611/aoe.2019.2.16

[ref81] Trąpczyński P Gorynia M Nowak J : How Does Economic Integration Affect Progress along the Investment Development Path? A Case Study of EU Member vs. Non-Member Countries from Eastern Europe. *Journal of Modern Science.* 2022;49(2):177–214. 10.13166/jms/155315

[ref82] United Nations: World Investment Report 2014, Overview - Investing in the SDGs, An Action Plan. *United Nations Conference on Trade and Development.* New York, and Geneva: United Nations;2014.

[ref83] Verma R Brennan L : The investment development path theory: evidence from India. *Int. J. Emerg. Mark.* 2011;6(1):74–89. 10.1108/17468801111104386

[ref84] Wardhani FS Haryanto T : Foreign Direct Investment in Agriculture and Food Security in Developing Countries. *Contemporary Economics.* 2020;14(4):510–521.

[ref85] Wooldridge JM : *Econometric Analysis of Cross Section and Panel Data.* Cambridge, MA: MIT Press;2002.

[ref86] World Bank: Agriculture and Food. 2022. Reference Source

[ref87] World Bank: DataBank, World Development Indicators. 2023a. Reference Source

[ref88] World Bank: DataBank, World Governance Indicators. 2023b. Reference Source

[ref91] Zhubikenov A : Kazakhstan and its investment development path. *Copernican Journal of Finance & Accounting.* 2022;11(3):85–97. 10.12775/CJFA.2022.015

