# Supplementary material for: The Investment development path theory: Evidence from developing countries’ agricultural sector
Source: F1000Res. 2024 Mar 8;12:1245. Originally published 2023 Sep 28. [Version 3] doi: 10.12688/f1000research.139491.3 (PMC11061594; doi:10.12688/f1000research.139491.3)
Supplement: Peer review history

# Reviewer report: Reviewer response for version 3

**DOI:** 10.5256/f1000research.163454.r255655

**Published:** 2024-05-08

**Authors:** Boban Dasic

**Affiliations:** 1 Kosovo and Metohija Academy of Applied Studies, Leposavić, Serbia

The work is very well done. In the paper, the authors examined the investment development path (IDP) from the perspective of developing countries' agricultural sector.

 The work takes into account interactions among countries regarding cross-border resource transfers.

 The stage of the IDP reflects the cross-border attractiveness of agriculture and tortuously of agricultural businesses going abroad from the agricultural sector and gives guideposts for apt economic strategy.

Is the work clearly and accurately presented and does it cite the current literature?

Yes

If applicable, is the statistical analysis and its interpretation appropriate?

Yes

Are all the source data underlying the results available to ensure full reproducibility?

Yes

Is the study design appropriate and is the work technically sound?

Yes

Are the conclusions drawn adequately supported by the results?

Yes

Are sufficient details of methods and analysis provided to allow replication by others?

Yes

Reviewer Expertise:

National economy, agricultural economy

I confirm that I have read this submission and believe that I have an appropriate level of expertise to confirm that it is of an acceptable scientific standard.

# Reviewer report: Reviewer response for version 3

**DOI:** 10.5256/f1000research.163454.r255665

**Published:** 2024-04-30

**Authors:** Bienmali Kombate

**Affiliations:** 1 School of Accounting, Guangzhou College of Technology and Business, Guangzhou, Guangdong, China

The work looks well structured and all the sections discussed the most appropriate concepts. The study has a sound novelty given its cross-sectional framework, data sources and the statistics models applied. 

 I only have minor suggestions

 I suggest that the corresponding author to consider changing the heading of the section three named “Data, Models and Modelling” to “Data and Method.”

 The study design subsection should be extended to add value. Try to explain why the choice of this method and what is it advantages and disadvantage grounding of the previous work. It is suggested to consider discussing the data and data sources under the subsection of “Research design.” The subsection heading named “Model” is suggested to change to be renamed “Methodology” and spare this subsection into two which discuss “the research model specification” and “the research variables”.

 Finally, prove the theoretical and practical implication of this work and the study limitations given the cross-sectional framework.

 Good luck and congratulations

Is the work clearly and accurately presented and does it cite the current literature?

Yes

If applicable, is the statistical analysis and its interpretation appropriate?

Yes

Are all the source data underlying the results available to ensure full reproducibility?

Yes

Is the study design appropriate and is the work technically sound?

Yes

Are the conclusions drawn adequately supported by the results?

Yes

Are sufficient details of methods and analysis provided to allow replication by others?

Yes

Reviewer Expertise:

Financial Risk Management

I confirm that I have read this submission and believe that I have an appropriate level of expertise to confirm that it is of an acceptable scientific standard.

# Reviewer report: Reviewer response for version 3

**DOI:** 10.5256/f1000research.163454.r254075

**Published:** 2024-04-10

**Authors:** Jorge Fleta-Asín, Marta Fernández-Olmos, Talía Gómez Aguas

**Affiliations:** 1 Business and Management, Universidad de Zaragoza, Zaragoza, Aragon, Spain

The current version reflects the limitations of the work. It is a work that provides insights into the Development Path Theory and its application.

Is the work clearly and accurately presented and does it cite the current literature?

Partly

If applicable, is the statistical analysis and its interpretation appropriate?

Yes

Are all the source data underlying the results available to ensure full reproducibility?

Yes

Is the study design appropriate and is the work technically sound?

Yes

Are the conclusions drawn adequately supported by the results?

Yes

Are sufficient details of methods and analysis provided to allow replication by others?

Yes

Reviewer Expertise:

Foreign Direct Investment, Public-Private Partnerships, Gender

We confirm that we have read this submission and believe that we have an appropriate level of expertise to confirm that it is of an acceptable scientific standard.

# Response

**Published:** 2024-04-10

**Authors:** Justice Djokoto

**Affiliations:** Central University, Ghana

We are most grateful for the comments and the 'approved' recommendation.

# Reviewer report: Reviewer response for version 2

**DOI:** 10.5256/f1000research.163092.r251183

**Published:** 2024-03-06

**Authors:** Jorge Fleta-Asín, Marta Fernández-Olmos, Talía Gómez Aguas

**Affiliations:** 1 Business and Management, Universidad de Zaragoza, Zaragoza, Aragon, Spain

The authors partially meet our requirements.

 We note that there is no mention of possible issues of multicollinearity with quadratic or cubic variables, nor is there an explanation as to why explanatory variables for the variable being estimated are not lagged by at least one year relative to the investment made. It is also suggested to provide a justification for studies analyzing the IDP in specific sectors and their validity beyond the entirety of the national economy. These are important points that could enhance the quality and validity of the study. Or at least the authors can mention them in the limitations section.

 Anyway, it was a pleasure to review it and we think that it is an interesting article.

Is the work clearly and accurately presented and does it cite the current literature?

Partly

If applicable, is the statistical analysis and its interpretation appropriate?

Yes

Are all the source data underlying the results available to ensure full reproducibility?

Yes

Is the study design appropriate and is the work technically sound?

Yes

Are the conclusions drawn adequately supported by the results?

Yes

Are sufficient details of methods and analysis provided to allow replication by others?

Yes

Reviewer Expertise:

Foreign Direct Investment, Public-Private Partnerships, Gender

We confirm that we have read this submission and believe that we have an appropriate level of expertise to confirm that it is of an acceptable scientific standard, however we have significant reservations, as outlined above.

# Response

**Published:** 2024-03-06

**Authors:** Justice Djokoto

**Affiliations:** Central University, Ghana

**Response to Jorge Fleta-Asin's comments on version 2**

 We thank Jorge Fleta-Asín for making time to review version 2 of the submission. We are also grateful for the valuable comments.

*Comment*

 The authors partially meet our requirements.

*Response*

 In what follows, we make additional revisions hoping that will meet your kind consideration.

*Comment*

 We note that there is no mention of possible issues of multicollinearity with quadratic or cubic variables, nor is there an explanation as to why explanatory variables for the variable being estimated are not lagged by at least one year relative to the investment made.

*Response*

 We acknowledge the role of multicollinearity in our response to the reviewer on version 1, but we did not update version 2. We now include the comment on multicollinearity in version 3. We create a last paragraph to the results section of version 3 as follows:

 “We acknowledge the role of multicollinearity especially when using powers of the same variable as explanation variables. An important consequence of multicollinearity is the inflation of the standard errors leading to the invalidation of the hypothesis tests. Our hypotheses tests are not appreciably invalidated, moreover, the estimates are robust and consistent with theory, thus, we did not consider the impairment of our results due to multicollinearity.”

   

 Regarding the lags, first, the dependent variable is not inward FDI only. It is outward FDI minus inward FDI. Thus, we are not modelling the location factor of inward FDI for which lags of the location factors may be relevant. Secondly, more than 100 published literature on the IDP does introduce lags explicitly. We do not intend to depart from that. Lags may, however, be relevant in cases where GMM estimation is required.

*Comment*

 It is also suggested to provide a justification for studies analyzing the IDP in specific sectors and their validity beyond the entirety of the national economy. These are important points that could enhance the quality and validity of the study. Or at least the authors can mention them in the limitations section.

*Response*

 In our response to the reviewer’s comment on version 1, we justified the plausibility of the sectoral (agriculture) IDP and updated the second paragraph of the introduction in version 2. We now do a further update of version 2, so the beginning of the second paragraph of version 3 reads as follows:

 “The investment development path is established by the interaction of net foreign direct investment and the gross domestic product per capita. Sectors of the economy possess net foreign direct investment and the gross domestic product per capita, so IDP can be established for sectors. Additionally, as the levels of these indicators can change, the IDP for each sector can change in ways that the IDP of the total economy can change. Further, Gorynia, Nowak and Wolniak (2009) demonstrated some sectoral perspectives on the IDP, including manufacturing. Hence, as in the case of the total economy, the IDP could apply to the agriculture sector which continues to be important for many developing countries. Indeed, as the agricultural sector has been studied across economies in the world, a similar study of the IDP in the agricultural sector is in place. The case of agriculture is interesting because the United Nations Food and Agricultural Organisation (FAO) has a dedicated website and database for agriculture with data including foreign direct investment and agricultural gross domestic product. Please see https://www.fao.org/faostat/en/#data/FDI”

 Following the justification, we do not consider the sectoral analysis a limitation.

*Comment*

 Anyway, it was a pleasure to review it and we think that it is an interesting article.

*Response*

 We are most grateful, and we will be glad if you can make time to review version 3.

# Reviewer report: Reviewer response for version 1

**DOI:** 10.5256/f1000research.152768.r243485

**Published:** 2024-02-20

**Authors:** Jorge Fleta-Asín, Talía Gómez Aguas, Marta Fernández-Olmos

**Affiliations:** 1 Business and Management, Universidad de Zaragoza, Zaragoza, Aragon, Spain

The document titled "Impact of Agricultural Trade Openness on the Investment Development Path for Agriculture in Developing Countries" explores the relationship between agricultural trade openness and the Investment Development Path (IDP) for agriculture in developing countries. The authors use data from 1991 to 2021 for 55 developing countries to empirically investigate the IDP, which indirectly considers interactions among countries regarding cross-border resource transfers. Through inferential statistics, the study aims to provide insights relevant for policymaking and economic strategy in the agricultural sector.

 The authors employ an analytical approach, incorporating random effects estimates and robustness checks with control variables to ascertain the impact of agricultural trade openness on agricultural net foreign direct investment (NOFDIPC). Their findings suggest that agricultural trade openness enhances NOFDIPC, while variables such as the exchange rate and GDP per capita exhibit opposite effects. Additionally, the study provides insights into the stages of the IDP, indicating that agricultural production in developing countries aligns with stages I and II of the IDP. Policy recommendations include fostering policies to increase both outward and inward foreign direct investment (OFDI and IFDI), developing institutional capacity for FDI, and promoting trade in agricultural commodities.

 We appreciate having been the reviewers of this article. We found the article interesting, it is well written, we have also learned and we believe there is room for improvement.

 Below are a series of comments to improve the work:

**1.** In the introduction it is said that there are works that analyze the IDP in a country (its path) or groups of countries, but they are not rigorous (they do not control for other variables) and do not focus on agriculture. This is not totally true because there are works where the PDI per se is analyzed or it is used with many control variables (in fact more and different than those used by the authors). An example here: Ref [1].

 Furthermore, I had not read that the IDP could explain isolated sectors, and I do not know to what extent it is a weakness of the work that we do not know if those countries with all the sectors would obtain the same results again (being in Stages I and II). We would have to be more cautious in the introduction, finding the gap.

**2.** The IDP figure should indicate whether it is from the authors, they have taken it from another article, or it is based on “X” article.

**3.** Regarding the methodology. While the authors employ rigorous statistical methods, enhancing transparency in the methodology section by providing more detailed explanations of the analytical approach, model specifications, and data sources would improve the reproducibility and credibility of the study. For example:

 - The NOFDIPC variable must be better justified. Why is it divided by the population? Why aren't logarithms applied?

 - Why are the control variables chosen and not others? And how do each of them influence each other specifically? Only a few are mentioned.

 - Doesn't the fact of introducing 3 variables that are combinations interacting with each other generate multicollinearity? The correlation table should appear, justifying its impact on the model (VIF), as well as considering the possibility of centering the interactions with respect to the means.

 - How are developing countries selected? Explain the criterion, because this can change and there are countries that over a period, if it is GDP/capita, can change their status. You should set the criteria and source. Are all the countries “developing” in such a long period of time according to one standard source?

 - Can you explain us how ((2021-1991)+1)* 55) countries makes you have around 800 observations?

 - The R ^2^ of the models is very low, which may be a sign that many other things are not being considered in those models. Maybe it would be good to explain it and put in limitations.

 - When an investment attraction variable is estimated, its explanatory factors (generally location factors) are usually delayed by at least one period, given that it is understood that what motivates the decision precedes the subsequent result. The models do not mention that they are delayed, it does not appear in the t-1 subscripts, nor is it explained why it is not done. Either it is justified, or it is done, or it is put in limitations.

 - At the end of Table 4 a number 3 appears at the bottom, which we do not know what it is.

 - In the notes of the tables you mention “parenthesis”, should it be “parentheses” once they are two?

**4.** About the contribution of the work and discussion/limitations.

 In the end, the conclusion is that developing countries, specifically in the agriculture sector, are located in stages I and II of development, something that would explain the theory and would not be adding anything new. The authors should “sell” the implications or singularities of the work better.

 While the study acknowledges its focus on developing countries, further discussion on the limitations and generalizability of the findings would strengthen the article. Addressing potential confounding variables or factors not accounted for in the analysis could enhance the robustness of the conclusions.

**5.** Future Research Directions.

 Including a section on future research directions could add value to the article by highlighting avenues for further exploration, such as examining the role of specific policy interventions or conducting comparative analyzes across different regions or country groups.

 Addressing these points would contribute to making the article scientifically sound and enhance its impact on both academic research and policymaking in the agricultural sector.

Is the work clearly and accurately presented and does it cite the current literature?

Partly

If applicable, is the statistical analysis and its interpretation appropriate?

Yes

Are all the source data underlying the results available to ensure full reproducibility?

Yes

Is the study design appropriate and is the work technically sound?

Yes

Are the conclusions drawn adequately supported by the results?

Yes

Are sufficient details of methods and analysis provided to allow replication by others?

Yes

Reviewer Expertise:

Foreign Direct Investment, Public-Private Partnerships, Gender

We confirm that we have read this submission and believe that we have an appropriate level of expertise to confirm that it is of an acceptable scientific standard, however we have significant reservations, as outlined above.

## References

[1] : Is the Importance of Location Factors Different Depending on the Degree of Development of the Country?. *Journal of International Management* .2016;22(1) : 10.1016/j.intman.2015.10.002 29-43 10.1016/j.intman.2015.10.002

# Response

**Published:** 2024-02-23

**Authors:** Justice Djokoto

**Affiliations:** Central University, Ghana

**Responses to Review by J. Fleta-Asin**

**Comments: Background**

 The document titled "Impact of Agricultural Trade Openness on the Investment Development Path for Agriculture in Developing Countries" explores the relationship between agricultural trade openness and the Investment Development Path (IDP) for agriculture in developing countries. The authors use data from 1991 to 2021 for 55 developing countries to empirically investigate the IDP, which indirectly considers interactions among countries regarding cross-border resource transfers. Through inferential statistics, the study aims to provide insights relevant to policymaking and economic strategy in the agricultural sector.

 The authors employ an analytical approach, incorporating random effects estimates and robustness checks with control variables to ascertain the impact of agricultural trade openness on agricultural net foreign direct investment (NOFDIPC). Their findings suggest that agricultural trade openness enhances NOFDIPC, while variables such as the exchange rate and GDP per capita exhibit opposite effects. Additionally, the study provides insights into the stages of the IDP, indicating that agricultural production in developing countries aligns with stages I and II of the IDP. Policy recommendations include fostering policies to increase both outward and inward foreign direct investment (OFDI and IFDI), developing institutional capacity for FDI, and promoting trade in agricultural commodities.

 We appreciate having been the reviewers of this article. We found the article interesting, it is well written, we have also learned and we believe there is room for improvement.

*Response*

 We acknowledge your summary and general impressions of the paper. However, we note that the title of the paper is “The Investment Development Path Theory: Evidence from developing countries’ agricultural sector”.

 Below are a series of comments to improve the work:

*Specific Comments*

 1. In the introduction it is said that there are works that analyze the IDP in a country (its path) or groups of countries, but they are not rigorous (they do not control for other variables) and do not focus on agriculture. This is not totally true because there are works where the PDI per se is analyzed or it is used with many control variables (in fact more and different than those used by the authors). An example here: Ref [1].

*Response*

 The reference: Ramirez-Aleson, M., & Fleta-Asin, J. (2016). Is the importance of location factors different depending on the degree of development of the country? *Journal of International Management, 22*(1), 29-43, addressed the location factors of FDI. The dependent variable is FDI and not NOFDIPC as in our paper. Although the countries analysed were claimed by Ramirez-Aleson and Fleta-Asin to be based on the IDP, this was not the case. The authors used GDP per capita only to categorise countries into stages of development. This is at variance with the IDP methodology developed by JH Dunning, which employed the interaction of the net FDI (outward FDI – inward FDI) and GDP per capita. Therefore, Ramirez-Aleson and Fleta-Asin (2016) is not an IDP study in the true sense of the IDP methodology. Although many control variables were used, that is irrelevant to the study under review. Therefore, our failure to acknowledge Ramirez-Aleson and Fleta-Asin (2016) is in place. Hence, the assertions of the authors of the study under review, are ‘totally’ true.

*Comment*

 Furthermore, I had not read that the IDP could explain isolated sectors, and I do not know to what extent it is a weakness of the work that we do not know if those countries with all the sectors would obtain the same results again (being in Stages I and II). We would have to be more cautious in the introduction, finding the gap.

*Response*

 The application of IDP to sectors has been in the literature since 2009. See Gorynia, M., Nowak, J., & Wolniak, R. (2009). Poland's Investment Development Path: in search of a synthesis. *International Journal of Economic Policy in Emerging Economies, 2*(2), 153-174. See especially Figure 3, where the IDP chart of various sectors including manufacturing have been captured using charts and not rigorous econometric analysis. Moreover, as the IDP is dependent on the interaction of the net FDI and the GDP per capita, which can be different for industries in the same country and can differ between sectors and the total economy, differences in IDP between the sector and the total economy are plausible. We are thankful for this comment as it suggests readers could have the same concern. Thus, we have updated the second paragraph of the introduction to address this matter.

*Comment*

 2. The IDP figure should indicate whether it is from the authors, they have taken it from another article, or it is based on “X” article.

*Response*

 The IDP figure is like others in some respects, but it is different from all others in its entirety.  All figures are from the authors; thus, no specific acknowledgements were made.

*Comments*

 3. Regarding the methodology. While the authors employ rigorous statistical methods, enhancing transparency in the methodology section by providing more detailed explanations of the analytical approach, model specifications, and data sources would improve the reproducibility and credibility of the study. For example:

*Response*

 Model specification is present, see for example equation 3. The data sources are outlined after equation 3. Additional information is provided to further enhance the paper.

*Comment*

 - The NOFDIPC variable must be better justified. Why is it divided by the population? Why aren't logarithms applied?

*Response*

 We have now explained that in the manuscript. We avoided logarithms as it would clear the nonlinearity that we seek to measure.

*Comment*

 - Why are the control variables chosen and not others? And how do each of them influence each other specifically? Only a few are mentioned.

*Response*

 We followed the literature in doing that (Andreff, 2003; Djokoto, 2021a; Durán and Ubeda, 2001, 2005; Frenken and Mbuvi, 2017). We focused less on the control variables are they are not the key variables.

*Comment*

 - Doesn't the fact of introducing 3 variables that are combinations interacting with each other generate multicollinearity? The correlation table should appear, justifying its impact on the model (VIF), as well as considering the possibility of centering the interactions with respect to the means.

*Response*

 We noted the VIF during our analysis. An important consequence of multicollinearity is the inflation of the standard errors leading to the invalidation of the hypothesis tests. Our hypothesis tests are not appreciably invalidated, moreover, the estimates are robust and consistent with theory, thus, we discounted the role of multicollinearity.   

*Comments*

 - How are developing countries selected? Explain the criterion, because this can change and there are countries that over a period, if it is GDP/capita, can change their status. You should set the criteria and source. Are all the countries “developing” in such a long period of time according to one standard source?

*Response*

 The developing countries were chosen based on the United Nations classification in the statistical annexe of World Economic Situation and Prospects 2022 ( https://www.un.org/development/desa/dpad/wp-content/uploads/sites/45/WESP2022_ANNEX.pdf). The classification has not changed throughout the study. This classification is not based on only GDP per capita, as in Ramirez-Aleson and Fleta-Asin (2016), but on other economic properties. All countries are developing according to the source, from 1991 to 2021.

*Comment*

 - Can you explain us how ((2021-1991)+1)* 55) countries makes you have around 800 observations?

*Response*

 Not every country had data over the period, hence we used an unbalanced panel. This is common with FDI data, especially, the outward FDI. Further, the lags resulted in some data attrition leading to differences in the number of observations listed in the model diagnostics section of some tables. 

*Comment*

 - The R2 of the models is very low, which may be a sign that many other things are not being considered in those models. Maybe it would be good to explain it and put in limitations.

*Comment*

 In the econometric literature, low R2 are a concern if the model will be used for forecasting. However, as we used the model for hypothesis testing of the coefficients, the low R2 is not a limitation of the model.

*Comment*

 - When an investment attraction variable is estimated, its explanatory factors (generally location factors) are usually delayed by at least one period, given that it is understood that what motivates the decision precedes the subsequent result. The models do not mention that they are delayed, it does not appear in the t-1 subscripts, nor is it explained why it is not done. Either it is justified, or it is done, or it is put in limitations.

*Response*

 In IDP studies, the lag is not necessarily the case. Where GMM is used this takes account of the lags. Hence, we avoided the lags.

*Comment*

 - At the end of Table 4 a number 3 appears at the bottom, which we do not know what it is.

*Response*

 The ‘3’ was left after the footnote was edited. This is now deleted.

*Comment*

 - In the notes of the tables you mention “parenthesis”, should it be “parentheses” once they are two?

*Responses*

 Noted and correction is done.

*Comment*

 4. About the contribution of the work and discussion/limitations.

 In the end, the conclusion is that developing countries, specifically in the agriculture sector, are located in stages I and II of development, something that would explain the theory and would not be adding anything new. The authors should “sell” the implications or singularities of the work better.

*Response*

 The primary goal of any IDP paper is to establish the stage of development based on the theory. That is our primary objective as well. After stating that, we noted the policy recommendations, as required in an applied economics paper. 

*Comments*

 While the study acknowledges its focus on developing countries, further discussion on the limitations and generalizability of the findings would strengthen the article. Addressing potential confounding variables or factors not accounted for in the analysis could enhance the robustness of the conclusions.

*Response*

 We employed variables that have been used in IDP studies. Indeed, some did not use these at all. We have added a sentence (long one though) to account for this in the last paragraph of the paper.

*Comment*

 5. Future Research Directions.

 Including a section on future research directions could add value to the article by highlighting avenues for further exploration, such as examining the role of specific policy interventions or conducting comparative analyzes across different regions or country groups.

 Addressing these points would contribute to making the article scientifically sound and enhance its impact on both academic research and policymaking in the agricultural sector.

*Response*

 Perhaps, as it was not designated as a section, you missed the content. The last paragraph of the paper presented the limitations and recommendations for further research. An additional sentence is now added to the paragraph to account for the need for generalisation and the findings to other sectors.
